# Utility of serial urinary cytology in the initial evaluation of the patient with microscopic hematuria

**DOI:** 10.1186/1471-2490-9-12

**Published:** 2009-09-10

**Authors:** Kogenta Nakamura, Ali Kasraeian, Kenneth A Iczkowski, Myron Chang, John Pendleton, Satoshi Anai, Charles J Rosser

**Affiliations:** 1 Division of Urology, The University of Florida, Jacksonville, Florida, USA; 2 Department of Pathology, The University of Florida, Gainesville, Florida, USA; 3 Department of Epidemiology and Health Policy Research, The University of Florida, Gainesville, Florida, USA; 4 Department of Urology, The University of Florida, Gainesville, Florida, USA

## Abstract

**Background:**

We determine the utility of serial urinary cytologies in patients presenting with microscopic hematuria who were evaluated with upper and lower urinary tract studies to rule out a malignancy.

**Methods:**

Two hundred and thirty-seven patients with the diagnosis of microscopic hematuria were evaluated at an inner-city tertiary care hospital. Of these 239 patients, 182 patients had 405 cytologies obtained as part of their evaluation for hematuria. In addition, all patients had their lower urinary tract and upper tract thoroughly evaluated.

**Results:**

Two hundred and seventy four cytology samples were read as normal, 104 (26%) as atypia, 7 (2%) as suspicious/malignant, and 20 (5%) as unsatisfactory. Seventeen patients (9.3%) had biopsy confirmed bladder cancer. Of these 17 patients, 2 had normal cytology, 11 had atypia, and 5 had suspicious/malignant. No patient had a positive cytology and a negative biopsy. Overall the number of hematuric patients harboring bladder cancer was small (7%). Cytology #1 detected 4 cases of cancer, cytology #2 detected an additional case and cytology #3 did not detect any additional cancers.

**Conclusion:**

Because of this low prevalence of bladder cancer in patients presenting with microscopic hematuria and the low sensitivity of detecting bladder cancers, the utility of urinary cytology in the initial evaluation of patients with hematuria may be minimal. The exact role of urinary cytology in the evaluation of hematuria is unknown.

## Background

Gross or microscopic hematuria [≥3 red blood cells (RBC) per high power field (hpf)] may be caused by numerous factors - urinary calculi, hematologic abnormalities, infection, trauma, tuberculosis, and tumor [1]. Some of these factors (e.g., tumor) may be life threatening. Thus prompt, thorough evaluation and treatment are needed. Currently, evaluation for the hematuric patient consists of inspecting the lower urinary tract by cystourethroscopy, inspecting the upper urinary tract with computed tomography scan of the abdomen and pelvis with and without intravenous contrast, ultrasonography or intravenous pyelogram (IVP), and obtaining a urine sample for cytologic evaluation [2,3].

Urine for cytology can detect cancerous cells shed from any part of the entire urothelium (i.e., collecting system to urethra) in a voided urine specimen. Higher grade tumors or larger tumors (> 3 cm) are more likely to shed cells into the urine and thus the sample is more likely to be positive for cancer. Urinary cytology has a notoriously low sensitivity, but an extremely high specificity, thus making it a useful tool in following patients with high grade cancers [4,5]. Furthermore, interpretation of urinary cytology may be difficult, especially in the face of such conditions as urinary tract infection. With these notable limitations, the question arises as to how effective is urinary cytology in diagnosing bladder cancer in a patient presenting with microscopic hematuria? Herein, we report the utility of urinary cytology of patients presenting to a urology clinic for evaluation of microscopic hematuria.

## Methods

### Study population

Institutional review board approval was obtained to query medical records for pertinent clinical information in 239 consecutive patients evaluated between January 2003 and August 2005 for microscopic hematuria at a urology outpatient clinic in a tertiary-care inner-city hospital. Microscopic hematuria was characterized as microscopic hematuria [≥ 3 red blood cells (RBC) per high power field (hpf)] [6]. Initial evaluations included medical history; physical examination, urinalysis, and voided urinary cytology. Urine cultures were obtained in patients if there was high suspicion of a urinary tract infection as the cause of the hematuria. Clinic and hospital records were reviewed for several key factors including tobacco usage, voiding symptoms (American Urologic Association, AUA, symptom score), urinalysis, urinary cytology, cystoscopic/radiologic evaluation, and pathologic outcomes. Median follow-up was 40 months (range 1-66 months).

Based on AUA guideline criteria on the use of urinary cytology, voided urinary cytologies were collected over a seven day period prior to radiologic and cystoscopic evaluation. Of the 239 patients, 182 patients had one cytology available for review whereas 125 and 96 patients had two and three cytologies, respectively, available for evaluation. Thus a total of 403 urinary cytologies were reviewed by our cytopathologists. In accordance with accepted nomenclature, final cytologic testing results were classified by the cytopathologists into 1 of 4 categories: normal, atypical/indeterminate, suspicious, or malignant [7].

### Cystoscopic evaluation of the lower urinary tract and bladder biopsy

Hematuria evaluation included cystourethroscopy performed by an attending urologist with a 30- and 70-degree endoscope in a 17-F sheath in women in the outpatient setting, an 18-F flexible cystoscopy in men in the outpatient setting, or with a 30-and 70-degree endoscope with a 21-F sheath in the operating room. Any abnormal bladder lesions were biopsied and frank tumors were resected. Pathologic specimens were sent for evaluation in 10% formalin. In patients with atypia, suspicious, or carcinoma on cytology and no obviously bladder tumor, random bladder and prostatic biopsies were obtained at the discretion of the treating surgeon.

### Radiologic evaluation of the upper urinary tract

The majority of patients (175, 74%) had their upper tracts evaluated by computed tomography urography scan of the abdomen and pelvis consisting of non-contrasted and contrasted images [8]. Intravenous pyelogram (IVP) was used to evaluate the upper tracts of 6 (3%) patients. In patients with an intravenous contrast allergy or another contraindication for CT scan or IVP, magnetic resonance imaging (n = 15, 6%) or renal ultrasound and retrograde pyelogram (n = 69, 29%) was used to evaluate the upper urinary tracts.

### Outcome Assessment

Biopsy specimens were graded histologically according to established grading systems [9,10]. The 2002 Tumor-Node-Metastasis (TNM) staging system was used for clinical staging [11].

### Statistical Analysis

Differences in distribution of demographic and clinical variables were evaluated using the Chi-square test or the Kruskal-Wallis test. P-values were obtained from the Fisher's exact test for testing the null hypothesis that there is no association between a "risk" factor (e.g., tobacco, voiding symptoms, cystoscopic or radiologic abnormality associated with bladder cancer) and abnormal urinary cytology vs. the one-sided alternative hypothesis that there is positive association between a "risk" factor and abnormal urinary cytology. P < 0.05 is significant. Statistical analysis was performed using SAS software (SAS Institute, Cary, North Carolina).

## Results

Of the 239 patients presenting for evaluation of microscopic hematuria, 182 patients obtained urinary cytology in the initial evaluation. Table 1 depicts the demographic and clinical characteristics of all 239 patients presenting for evaluation of microscopic hematuria.

**Table 1:** Demographics and clinical features of 239 patients presenting for microscopic hematuria evaluation

**Characteristic**	**No cytology available**		**Cytology available**	
	**N = 57**		**N = 182**	

**Age**	(N)	(%)	(N)	(%)

**Range**	18-91		20-89	

**Median**	63		60	

**Sex**				

**M/F**	36/21	63/37	92/90	51/49*

**Race**				

**Caucasian**	35	61	113	62

**African American**	16	29	59	32

**Other**	6	11	10	6

**Tobacco use**	22	39	110	60*

* difference between patients with cytology available compared to patients without cytology, *p *< 0.05

A total of 403 cytologic specimens were reviewed. Eighteen samples were unsatisfactory for interpretation due to scant number of cells or severe degradation. Thus 385 specimens were reported. Two hundred and seventy four urinary cytology samples were classified as negative (72%) and 104 (27%) as atypia. The remaining 7 urinary cytologies from 5 patients were categorized as suspicious or malignant, which accounted for 1% of all of the urinary cytology evaluated. Cytology #1 detected 4 cancers and cytology #2 detected an additional cancer case (confirmed 2 cytologies from #1) whereas cytology #3 did not detect any additional cancers.

Of the 182 patients with available cytologic data, 38 (21%) demonstrated an abnormality on cystoscopic examination worrisome for malignancy, 9 (5%) an abnormality on radiologic evaluation worrisome for malignancy, or 10 (5%) an abnormality on both worrisome for malignancy. Table 2 illustrates both cytologic and cystoscopic findings of the 182 patients. None of the non-suspicious bladder tumors proved to be cancer and 17 (45%) of the suspicious bladder tumors were found to be malignancy (Table 3). Biopsy proven bladder cancer was evident in 2% of patients with normal cytology, 15% of patients with atypia, and 100% of patients with suspicious or malignant cytology (Table 4). None of the patients with biopsy proven bladder cancer was found to have carcinoma in situ. Combining the above two groups (atypia and suspicious/malignant cytology), we determined the unadjusted odds ratio (ORs) with 95% CIs for patient characteristics that are associated with atypical or suspicious/malignant cytology (Table 5).

**Table 2:** Cytologic and cystoscopic results of 182 patients presenting with microscopic hematuria

**Test result**	**N (%)**
**Cytology**	

Negative*	103 (57%)

Suspicious/Positive	5 (3%)

Atypia	74 (41%)

**Cystoscopy**	

Normal	115 (63%)

Non-suspicious Bladder Mass	5 (3%)

Suspicious Bladder Mass	38 (21%)

others**	24 (13%)

*: includes reactive changes, degenerative changes

** included stones, cystitis

**Table 3:** Comparison of cystoscopic findings with biopsy pathology

	**Cystoscopy data**		
**Biopsy results**	Normal* (N = 144)	**Benign lesion (N = 5)**	**Tumors** **(N = 38)**

**Positive for cancer** **(N = 17)**	0	0	17

**Negative for cancer** **(N = 70)**	49	5	21

* not all patients with normal cystopscopy underwent bladder biopsy.

**Table 4:** Comparison of cytologic findings with final pathology

	**Cytologic diagnosis^#^**		
**Biopsy results**	Negative * (N = 103)	Atypical* (N = 74)	**Suspicious/Positive** **(N = 5)**

**Positive for cancer** **(N = 17)**	2	11	5

**Negative for cancer** **(N = 70)**	47	23	0

# most adverse cytologic result per patient

*not all patients underwent bladder biopsy.

**Table 5:** Risk factors for abnormal (atypical, suspicious, or malignant) urinary cytologic testing

**Characteristic**		**Risk of Abnormal Cytology (m/n)**	**Unadjusted OR** **(95% CI)**	**P-Value**
Tobacco	Smoker	48/109 (44%)	0.98 (0.53-1.81)	0.59
			
	Non-Smoker	29/65 (45%)		

Dysuria	Yes	12/31 (39%)	0.77 (0.35-1.70)	0.80
			
	No	68/151 (68%)		

UA	Positive	30/77 (39%)	0.82 (0.41-1.66)	0.76
			
	Negative	24/55 (44%)		

Radiology Study	Positive	10/16 (63%)	2.29 (0.79-6.58)	0.097
			
	Negative	70/166 (42%)		

None of the 23 patients with atypia and negative evaluation were noted to harbor symptoms or signs of bladder cancer on subsequent follow-up. No patient with a normal cystoscopic evaluation and abnormal cytology was noted to have bladder cancer on extensive evaluation. In addition, no patient had a positive cytology and a negative biopsy. Median follow-up was 40 months with no patient with a negative cytology and/or negative bladder biopsy diagnosed with subsequent cancer.

## Discussion

Our study indicates that the prevalence of bladder cancer in patients presenting with microscopic hematuria is low (7%). A key question is what is the proportion of subjects in whom a positive cytology prompted a biopsy that was positive for cancer, in whom a biopsy would not otherwise have been performed? None of our patients had a positive cytology and a negative cytsoscopic/radiologic evaluation. This is consistent with our previous study that demonstrated an extremely low yield of urinary cytology in the evaluation of the patient with microscopic hematuria [12]. To our knowledge, these are the first contemporary report bringing the utility of urinary cytology in patients with microscopic hematuria into question.

As for the application of serial urinary cytologies, previous studies demonstrated a marginally improved by serial examinations [4,13], which was confirmed in our study. Because of this low prevalence and the low sensitivity, the utility of urinary cytology in the initial evaluation of patients with microscopic hematuria is minimal, especially since all high risk patients proceeded to cystourethroscopy and upper urinary tract radiologic evaluation.

Current AUA guidelines recommend that patients presenting with microscopic hematuria should undergo upper tract evaluation along with cystoscopy. When the CT scan is not feasible, MRI or renal ultrasound with bilateral retrograde pyelograms can be substituted (2). This evaluation is adequate in assessing the kidneys, collecting system, ureters, bladder, and urethra as the cause of the hematuria. Unfortunately, except in select patients our current diagnostic modalities will not allow us to diagnose urothelial carcinomas without visualization of a lesion followed by biopsy, which is the gold standard [14]. With this said, the acquisition of urinary cytology even as an adjunct to the above studies rarely changes the evaluation or management and may lead to an exhaustive, unfruitful, and costly evaluation. In the face of abnormal cytology and normal cystoscopy confirmed by biopsy and normal imaging of the upper tract, the question arises whether to pursue the abnormal cytology as a possible upper tract tumor. Evaluation may include retrograde pyelogram, ureteroscopy, and selective cytology. The yield of these maneuvers is reported to be extremely low and of little benefit, except in highly select patients [15,16], especially in the face of normal radiologic studies and normal cystoscopic evaluation. However, if an abnormality is noted on radiologic or cystoscopic examination, urinary cytology may prove to be useful as a confirmation of a malignancy prior to formal biopsy.

Recent studies have reported the limitations of urinary cytology in the evaluation of patients with hematuria. Paez and colleagues reported that no tumor could be diagnosed with cytology alone and that a negative cytology could not exclude a malignancy [17]. Because of its limitations, Nabi et al. recommended the judicial use of cytology in the proper clinical context [18]. Similar to the report by Deshpande et al., over 25% of patients with atypical urinary cytology were found to have biopsy proven cancer [19]. Because of this, atypia may require biopsy to rule out malignancy, closer follow-up, or other urinary based assays to improve sensitivity (e.g., fluorescent in situ hybridization, FISH).

Our study has several limitations. First, this is a small, retrospective study from a single institution. Not only could biases have been introduced in patient selection and evaluation, this group may not represent patients with microscopic hematuria seen by urologists outside of a tertiary care setting or those seen by primary care physicians. Secondly, a paucity of outside medical records were available to review in order to determine how the patients initially were found to have microscopic hematuria (i.e., were they diagnosed based on history, urine dipstick, microscopic analysis). Thirdly, other urine based assays (e.g., NMP-22, BTA, etc) also have reduced sensitivity in this cohort. Lastly, there was no standardized follow-up protocol in place to monitor patients with a negative hematuria evaluation in order to determine possible long-term developments.

The interpretation of urinary cytology can be extremely challenging and should be used only as an adjunct to evaluation of upper tract and bladder. Due to the complex nature of evaluating cytologic specimens, it is of utmost importance to have an experienced cytopathologist interpreting these results. Current urine based assays (e.g., NMP-22, BTA) are not at the point of being able to exonerate the bladder of harboring bladder cancer, thus patients suspected of a bladder cancer should be evaluated with upper tract imaging and cystoscopy. The addition of urinary cytology in patients with gross hematuria may be justifiable, however, its addition to the evaluation of the patient with microscopic hematuria has an extremely low yield in detecting cancer and may lead to unnecessary, invasive procedures with known side effects not to mention high costs.

## Conclusion

Current practice patterns for the evaluation of microscopic hematuria include assessing the lower and upper urinary tracts. Due to the low prevalence of bladder cancer and the low sensitivity of cytology detecting bladder cancer, the exact role of urinary cytology in the evaluation of the patient with microscopic hematuria may be minimal.

## Competing interests

Dr. Charles J. Rosser, Xceed Inc research scholarship to study bladder cancer. All other authors declare that they do not have a competing interests.

## Authors' contributions

KN, AK, JP, SA-Authors queried hospital records, formulated database and wrote manuscript.

KAI-Staff pathologist who assisted in the interpretation of results

MC-Participated in the design of the study and performed the statistical analysis

CJR-Conceived of the study, and participated in its design and coordination

All authors read and approved the final manuscript

## Pre-publication history

The pre-publication history for this paper can be accessed here:


